# The Vital Force Defined, by the Late Edward Bayard, M. D.

**Published:** 1892-02

**Authors:** Clarence C. Howard

**Affiliations:** 64 West 51st St., N. Y. City


### THE VITAL FORCE DEFINED.

By the late Edward Bayard, M. D., New York.

*Equalization is Health.* The vital force comes from God; it is a flow. The brain is covered with nerve cells. The vital force enters these cells and flows thence to the extremities.

Each portion of every organ by this flow is operated upon to the performance of those functions for which it was designed by the Creator.

Swedenborg says: "Of man's interior or mental elevation, however, this also ought to be understood. In everything created by God there is reaction. Action belongs to life alone, and reaction is caused by the action of life."

"Now because this reaction takes place whenever any created thing is acted upon, it seems to belong to the thing created; thus, in man, reaction seems to be his own because he has no sense of life but his own, although he is merely a recipient of life. Therefore it is, that man from his hereditary evils, reacts against God; but if he believes that all his life is from God, and all the good in life is from God's action, then his reaction becomes the result of the Divine action upon him, and he acts with God as from himself. By simultaneous action and reaction all things are held in equilibrium; and all things must be so held. This is here stated lest any man should believe that he ascends to God by his own power, and not by the power of the Lord." (Swedenborg's *Divine Love and Wisdom*, Philada. Edition, 1868, page 48, Sec. 68.)

"Consumption is an illustration of hereditary evil preventing the reaction from completing itself into equalization.

"The great thing to accomplish in order to promote health is *to restore equalization to all parts of the system.*

"This flow of vital force should reach every part. Not only must it reach every part, however minute, but there must be the unobstructed return current or *reaction* which is the cause of equalization. Obstruction in any part of this circuit of current flowing—whether occurring in the direct action or in the reaction of the vital force is the cause of pain, danger, and death; while equalization, as said before, is life and health.

"Each function can be performed only by its appropriate organ, and by the vital current furnishing that organ with the necessary flow of life for its operation.

"For example; bile can only be secreted by a liver, in the parts of which the vital force passes in the normal ways; sight can only be enjoyed by an eye supplied by the same force.

"This is as true as that no steam power can be utilized without a proper apparatus properly supplied with steam."

CLARENCE C. HOWARD.

64 WEST 51ST ST., N. Y. CITY.

